# The Delta Neutrophil Index as a predictive marker of histological chorioamnionitis in patients with preterm premature rupture of membranes: A retrospective study

**DOI:** 10.1371/journal.pone.0173382

**Published:** 2017-03-09

**Authors:** Hee Young Cho, Inkyung Jung, Ja-Young Kwon, So Jung Kim, Yong Won Park, Young-Han Kim

**Affiliations:** 1 Department of Obstetrics and Gynecology, Institute of Women’s Life Medical Science, Yonsei University College of Medicine, Yonsei University Health System, Seoul, Korea; 2 Department of Obstetrics and Gynecology, CHA Bundang Medical Center, CHA University, Seongnam, Korea; 3 Department of Biostatistics and Medical Informatics, Yonsei University College of Medicine, Seoul, Korea; 4 Department of Medicine, Yonsei University College of Medicine, Seoul, Korea; Ohio State University, UNITED STATES

## Abstract

**Background:**

Histological chorioamnionitis (HCA) is related to perinatal morbidity. However, there is no definite diagnostic method for detecting chorioamnionitis before delivery.

**Methods:**

We evaluated whether the delta neutrophil index (DNI) was an effective early marker of HCA in patients with preterm premature rupture of membranes (PPROM). We retrospectively evaluated 149 women diagnosed with PPROM (gestational age, 20^+0^ to 36^+6^ weeks) at Severance Hospital from January 2013 to December 2014. The women were categorized into the following two groups: (a) PPROM without HCA and (b) PPROM with HCA. The maternal white blood cell (WBC) count, neutrophil-to-lymphocyte ratio (NLR), C-reactive protein (CRP) level, and DNI were measured at admission. The DNI has been reported to reflect the fraction of circulating immature granulocytes associated with infection.

**Results:**

Of the 149 patients, 87 were included in the PPROM without HCA group and 62 were included in the PPROM with HCA group. The interval between admission and delivery was significantly shorter in the PPROM with HCA group than in the PPROM without HCA group. There was no significant difference in the maternal WBC count. The serum CRP level, NLR, and DNI were significantly lower in the PPROM without HCA group than in the PPROM with HCA group, while the lymphocyte count was significantly lower in the PPROM with HCA group than in the PPROM without HCA group. A predictive equation was generated by combining the DNI, lymphocyte count, and CRP level, and the sensitivity and specificity for predicting a placental inflammatory response were 69.1% and 70.5%, respectively.

**Conclusions:**

The DNI could be a predictive marker for HCA in patients with PPROM. Our predictive equation involving the DNI, lymphocyte count, and CRP level may be helpful for predicting the placental inflammatory response in patients with PPROM.

## Introduction

Preterm premature rupture of membranes (PPROM) is one of the leading causes of preterm birth [1], and approximately 30–40% of pregnant women with PPROM show intra-amniotic infection [2, 3]. Pregnant women with PPROM combined with intra-amniotic infection are at increased risk of adverse maternal and neonatal outcomes, such as preterm delivery, neonatal intraventricular hemorrhage, cerebral palsy, bronchopulmonary dysplasia, neonatal sepsis, and death [4, 5]. Intrauterine infection is one of the important risk factors for subsequent spontaneous preterm labor [6], and considering placental pathologic findings, the presence of intra-amniotic infection represents histological chorioamnionitis (HCA). A fetal inflammatory response occurs when there is infection of the umbilical cord, including the umbilical veins, umbilical arteries, and Wharton’s jelly [7]. It can be defined as funisitis and is related to a more advanced stage of intrauterine infection. However, intra-amniotic infection could be subclinical, and a microbiological study is required to confirm infection in the amniotic fluid. Gram staining and measurements of the white blood cell (WBC) count and glucose level in the amniotic fluid are the most sensitive and specific methods for detecting intra-amniotic infection. Despite the advantage of amniocentesis, it is an invasive procedure that can result in complications; thus, selection of this procedure needs careful consideration. Moreover, amniocentesis is impossible or difficult to perform in pregnant women with PPROM because of their decreased amniotic fluid volume. Previous studies reported that the success rate for obtaining amniotic fluid was 45–97% in patients with PPROM, and the results were primarily dependent on the condition of the accessible amniotic fluid pocket [8].

Recent studies have shown that the serum delta neutrophil index (DNI) is associated with diverse infectious conditions, such as bacterial peritonitis, acute appendicitis, and sepsis [9–11]. The DNI is a new inflammatory marker, but no recent studies have reported its predictive value in patients with PPROM with HCA.

Therefore, we conducted this study to determine whether the DNI is an effective early marker of HCA in patients with PPROM and to develop a non-invasive, easy, and prompt predictive equation that combined maternal serum markers and the DNI.

## Materials and methods

A total of 149 pregnant women with PPROM between 20^+0^ and 36^+6^ gestational weeks from January 2013 to December 2014 at Severance Hospital in Seoul, Korea were enrolled in this study. This study was approved by the Institutional Review Board of the Yonsei University Health System. We excluded women with multiple pregnancies, fetal anomalies, missing placental pathologic results, and medically indicated preterm birth. Additionally, pregnant women with hematologic or any other autoimmune disease affecting hematologic parameters were excluded.

Clinical data included demographic variables, such as maternal age; body mass index (BMI); preterm birth history; gestational age at diagnosis; and maternal WBC count, neutrophil count, lymphocyte count, neutrophil-to-lymphocyte ratio (NLR), C-reactive protein (CRP) level, and DNI. The diagnosis of PROM was based on clinical visual examination of amniotic fluid pooling in the vaginal cavity during sterile speculum examination or a positive nitrazine test. If the nitrazine test result was inconclusive, we performed the placental alpha microglobulin-1 test (AmniSure test). Sterile cotton swabs were used to obtain vaginal secretion samples for culture in order to assess group B streptococcus (GBS), *Ureaplasma urealyticum*, and *Mycoplasma hominis*. The serum CRP levels were determined using an automated nephelometer (Beckman Coulter Image, Fullerton, CA, USA) according to the manufacturer’s instructions. The detection limit of this analysis was 0.01 mg/dL, and the normal serum CRP level was considered to be <0.8 mg/dL [12, 13]. The serum DNI value was obtained using an automated cell analyzer (ADVIA 2120 Hematology System, Siemens, Healthcare Diagnostics, Forchheim, Germany). This is a flow cytometry-based hematologic analyzer, which uses two independent WBC counting methods, including a myeloperoxidase (MPO) channel and a lobularity/nuclear density channel. The DNI value was calculated using the following formula: DNI (%) = (the leukocyte subfraction assayed in the MPO channel by cytochemical reactions)–(the leukocyte subfraction assayed in the nuclear lobularity channel by reflected light beam measurements) [9, 14, 15]. Determination of the serum CRP level requires additional laboratory processes and expense, whereas the serum DNI value can be obtained during routine complete blood counts at our institution, without additional time or expense.

Placentas were sent to the pathologic department for detection of infection. Histopathologic chorioamnionitis was defined as microscopic evidence of infection on any part of the placenta, and deciduitis was defined as infection of the decidua. Funisitis was defined as infection of the connective tissue of the umbilical cord [16].

All pregnant women with PPROM included in this study were divided into the following two groups according to their pathologic results: (a) patients with PPROM without HCA and (b) patients with PPROM with HCA. All patients with PPROM received prophylactic intravenous antibiotics and 12 mg intramuscular betamethasone injection twice at a 24-hour interval for lung maturation. Tocolysis was used if patients had preterm labor. All pregnant women were treated until 34 gestational weeks if they showed no signs of clinical chorioamnionitis. If clinical chorioamnionitis occurred, delivery was performed.

Continuous variables are expressed as means and standard deviations (SDs) or medians and ranges. The chi-squared test or Fisher’s exact test was used for categorical variables, and the two-sample *t*-test or Wilcoxon rank sum test was used for continuous variables. Multiple logistic regression analysis was performed to estimate the odds ratio of HCA prediction with adjustment for confounders. The combination of significant parameters was used to generate a predictive equation. All statistical analyses were performed using SAS version 9.2 (SAS Institute, Inc., Cary, NC). Statistical significance was set at a *p*-value <0.05.

## Results

The study included 149 pregnant women who had PPROM between 20^+0^ and 36^+6^ gestational weeks. Of the 149 patients, 87 were included in the PPROM without HCA group and 62 were included in the PPROM with HCA group, and we compared the clinical characteristics and perinatal outcomes between these groups (Table 1). There were significant differences in gestational age at admission, gestational age at birth, interval between admission and birth, and cervix length at admission between the two groups. The cervix length at admission was significantly longer, while the interval between admission and birth was significantly shorter in the PPROM with HCA group than in the PPROM without HCA group (*p* = 0.004 and *p* = 0.005, respectively).

**Table 1:** Clinical characteristics and pregnancy outcomes in the PPROM without HCA and PPROM with HCA groups.

Variables	PPROM without HCA (n = 87)	PPROM with HCA (n = 62)	*p*-value
Maternal age (yrs)	33.4 ± 3.4	32.8 ± 3.3	0.299
Primiparous	44 (50.6%)	21 (33.9%)	0.221
Prior abortion	29 (33.3%)	26 (41.9%)	0.571
Previous PTB history	3 (3.5%)	5 (8.1%)	0.278
BMI at admission (kg/m^2^)	25.3 ± 3.6	24.7 ± 3.4	0.244
Gestational age at admission (wks)	33^+2^ (20^+3^–36^+6^)	31^+4^(20^+0^–36^+3^)	0.038
Gestational age at birth (wks)	33^+4^ (20^+4^–36^+6^)	31^+5^(20^+1^–39^+3^)	0.017
Interval of admission to birth (days)	4.4 ± 8.4	3.0 ± 8.5	0.005
Clinical chorioamnionitis	2 (2.3%)	3 (4.8%)	0.650
Cervix length (cm)	1.6 ± 1.3	2.4 ± 1.2	0.004
Delivery mode			0.006
Vaginal delivery	24 (27.6%)	31 (50%)	
Cesarean section	63 (72.4%)	31 (50%)	
Neonatal birth weight (g)	1879 ± 521	1735 ± 678	0.164
APGAR score at 1min	5 (0–8)	4 (0–7)	0.382
APGAR score at 5min	7 (0–9)	6 (0–9)	0.702
NICU admission (%)	82 (94.3%)	55 (88.7%)	0.238

PPROM, preterm premature rupture of membranes; HCA, histological chorioamnionitis; PTB, preterm birth; BMI, body mass index; NICU, neonatal intensive care unit

The distribution of the placental pathologic findings among the study patients is shown in Table 2. Among the 149 pregnant women, 62 (41.6%) had HCA, including chorioamnionitis, chorionitis, amnionitis, choriodeciduitis, and funisitis. The most common pathologic result was chorioamnionitis (39/62, 62.9%).

**Table 2:** Distribution of placental pathologic results.

**Pathologic results**	
Chorionitis	7 (11.3%)
Chorionitis + deciduitis	1 (1.6%)
Deciduitis	7 (11.3%)
Amnionitis + deciduitis	2 (3.2%)
Chorioamnionitis	39 (62.9%)
Chorioamnionitis + funisitis	3 (4.9%)
Chorionitis + deciduitis + funisitis	2 (3.2%)
Chorionitis + funisitis	1 (1.6%)
	62 (100%)

To evaluate the differences in laboratory variables between the PPROM without HCA and PPROM with HCA groups, we compared the laboratory results, including vaginal culture findings, between the two groups (Table 3). There were significant differences in the hemoglobin (Hb) levels, hematocrit (Hct) values, lymphocyte counts, NLRs, DNI values, and CRP levels between the two groups. The median NLRs were 4.7 (range, 2.3–17.9) and 6.2 (2.0–48.8) (*p* = 0.001), DNI values were 0 (0–2.6) and 0.25 (0–4.7) (*p* = 0.006), and CRP levels were 3.3 (0.6–32.0) and 7.5 (0.6–65.2) (*p* = 0.008) in the PPROM with HCA group and PPROM without HCA group, respectively. The NLR, DNI, and CRP levels were significantly higher, while the Hb levels, Hct values, and lymphocyte counts were significantly lower in the PPROM with HCA group than in the PPROM without HCA group. The median neutrophil count was higher in the PPROM with HCA group than in the PPROM without HCA group; however, the difference was not statistically significant. Among the study population, the proportion of patients with positive culture results for *Ureaplasma urealyticum* was higher in the PPROM with HCA group than in the PPROM without HCA group (37.9% [33/87] vs. 56.5% [35/62]; *p* = 0.034). However, there was no difference in the proportion of patients with positive culture results for *Mycoplasma hominis* between the two groups (*p* = 0.102).

**Table 3:** Laboratory characteristics of the PPROM without HCA and PPROM with HCA groups.

Variables	PPROM without HCA (n = 87)	PPROM with HCA (n = 62)	*p*-value
**Laboratory variables**			
Hemoglobin (g/dl)	12.08 ± 1.38	11.62 ± 1.13	0.034
Hematocrit	35.77 ± 4.23	34.33 ± 3.78	0.033
Platelet (x10^3^/L)	221.94 ± 66.20	226.3 ± 71.6	0.704
WBC (cells/L)	10.68 ± 3.91	11.30 ± 3.74	0.333
Neutrophil (cells/L)	7.57 (3.05–22.62)	8.67 (3.42–23.41)	0.114
Lymphocyte (cells/L)	1.6 (0.5–5.2)	1.4 (0.4–2.6)	0.016
Monocyte (cells/L)	0.49 (0.06–1.15)	0.48 (0.12–0.81)	0.508
Eosinophil (cells/L)	0.09 (0.01–0.45)	0.09 (0.01–0.39)	0.940
Basophil (cells/L)	0.03 (0–0.10)	0.03 (0–0.08)	0.334
PT (INR)	0.86 ± 0.06	0.88± 0.06	0.017
PTT (sec)	28.54 ± 2.91	28.30 ± 2.40	0.596
NLR	4.7 (2.3–17.9)	6.2 (2.0–48.8)	0.001
DNI (%)	0 (0–2.6)	0.25 (0–4.7)	0.006
CRP (mg/dl)	3.3 (0.6–32.0)	7.5 (0.6–65.2)	0.008
**Vaginal cultures**			
*Ureaplasma urealyticum*	33 (37.9%)	35 (56.5%)	0.034
*Mycoplasma hominis*	1 (1.2%)	1 (1.6%)	0.102

PPROM, preterm premature rupture of membranes; HCA, histological chorioamnionitis; WBC, white blood cell; PT (INR), prothrombin time (international normalized ratio); PTT, partial thromboplastin time; NLR, neutrophil-to-lymphocyte ratio; DNI, delta neutrophil index; CRP, C-reactive protein

**Fig 1:**
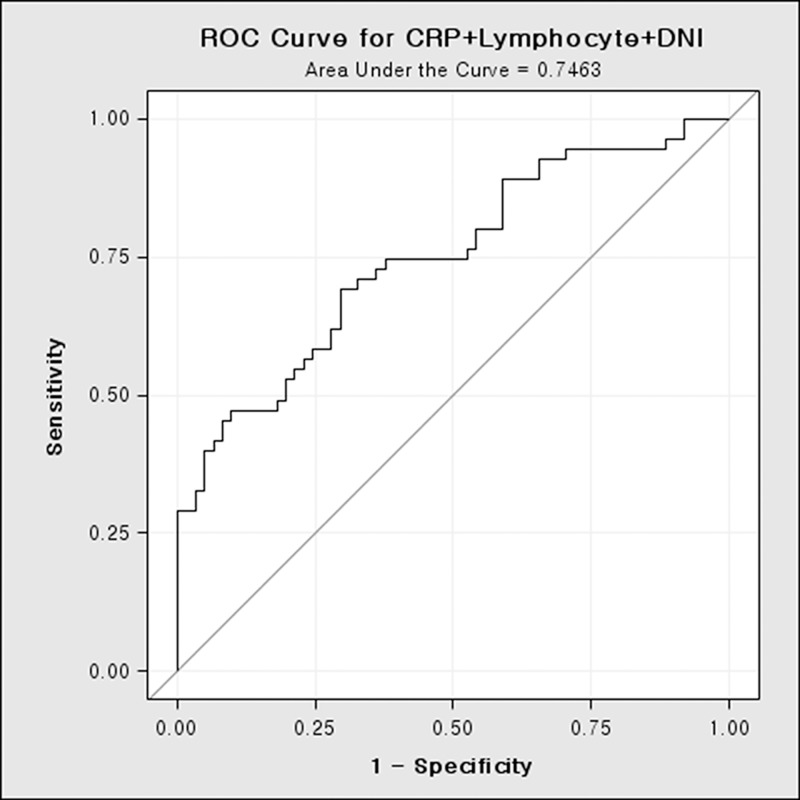
Receiver operating characteristic curve. Receiver operating characteristic curve for the prediction of chorioamnionitis using the DNI, CRP level, and lymphocyte count DNI, delta neutrophil index; CRP, C-reactive protein

In order to identify the value of the CRP level, lymphocyte count, and DNI that predicted HCA, we determined the odds ratios after adjusting for confounding factors (Table 4). The odds ratios for predicting HCA were 1.069 (95% CI: 1.017–1.125, *p* = 0.009) for the CRP level, 0.348 (95% CI: 0.157–0.773, *p* = 0.009) for the lymphocyte count, and 1.818 (95% CI: 1.104–2.991, *p* = 0.018) for the DNI. Increases in the CRP level and DNI were significantly associated with HCA, while decreases in the lymphocyte count were significantly associated with HCA. The combination of the CRP level, DNI, and lymphocyte count yielded the greatest area under the receiver operating characteristic curve (0.746) (Fig 1).

**Table 4:** Predictors of HCA as determined by logistic regression analysis.

Outcomes	OR	95% CI	*p*-value
CRP	1.069	1.017–1.125	0.009
Lymphocyte	0.348	0.157–0.773	0.009
DNI	1.818	1.104–2.991	0.018

HCA, histological chorioamnionitis; CRP, C-reactive protein; DNI, delta neutrophil index

A predictive equation was generated using these three parameters through logistic regression modeling. The estimated regression equation is defined as p = e^f^ / (1 + e^f^), where f is a linear function of the selected parameters and e is the base associated with the natural logarithm. Integration of the parameters yields the following equation:

$$p\;(Y=1)=\frac{exp(0.6434+0.0670\times CRP-1.0555\times lymphocyte+0.5975\times DNI)}{1+exp(0.6434+0.0670\times CRP-1.0555\times lymphocyte+0.5975\times DNI)}$$

When the predictive probability cutoff was 0.447 according to the Youden index, the sensitivity and specificity for predicting HCA were 69.1% and 70.5%, respectively.

## Discussion

To our knowledge, this is the first study to investigate the usefulness of the DNI as a predictive marker for HCA in patients with PPROM. We found that the DNI was a useful non-invasive marker for detecting HCA in pregnant women with PPROM. Moreover, we identified a predictive equation for HCA derived from the combination of the CRP level, lymphocyte count, and DNI, which was more accurate than was the use of each factor alone. The predictive equation for HCA can be used for prenatal counseling and management planning in patients with PPROM.

Intrauterine infection can be diagnosed based on amniocentesis; however, this approach is invasive and can result in complications. Recent studies have indicated that sterile intra-amniotic inflammation, which does not involve microorganisms, is highly associated with HCA [17, 18]. Roberts et al. [19] demonstrated that acute chorioamnionitis can occur without demonstrable intra-amniotic infection. In short, the diagnosis of intra-amniotic infection can be overlooked despite amniocentesis. On the other hand, HCA can be diagnosed through histologic examination of the placenta during the postpartum period. Therefore, these methods are inappropriate for detecting intrauterine inflammation in the prenatal period.

Many researchers have suggested different combinations of biomarkers for predicting intrauterine infection. Ryu et al. [20] showed that the combination of IL-6 in cervicovaginal fluid and gestational age at sampling was a useful predictive marker for intra-amniotic inflammation. However, this finding is difficult to apply in clinical settings because the measurement of IL-6 is time-consuming and requires a special detection kit. Other previous studies suggested the combination of the NLR and cervix length as a more sensitive marker for the prediction of preterm birth [21]. In this study, increased CRP levels and DNI values and decreased lymphocyte counts showed significant associations with HCA. A non-invasive predictive equation model was created using these three markers, and this model can be easily used to diagnose HCA during the prenatal period. Its sensitivity and specificity for predicting HCA were 69.1% and 70.5%, respectively. Regardless of the type of inflammation-causing microorganisms in HCA, the proinflammatory cytokines that are secreted in the early stage of intrauterine inflammation, such as interleukin (IL)-6, IL-10, IL-1β, and tumor necrosis factor-α (TNF-α), flow into the bloodstream of pregnant women. These changes affect the number of leukocyte subtypes and eventually cause neutrophilia [22]. Moreover, it has been reported that the TNF family that goes into action during the early stage of inflammatory response increases lymphocyte apoptosis, and the production of immunosuppressive factors significantly decreases the number of T-helper lymphocytes [23, 24].

In several studies, immature granulocytes have been investigated as predictors of sepsis because the number of immature granulocytes is known to increase in inflammatory conditions [25, 26]. However, there is no method to measure immature granulocytes accurately, and it is difficult to use conventional detection tests in infectious conditions. Nahm et al. [27] reported a study on the DNI based on automated immature granulocyte counts for determining the severity of sepsis to overcome this limitation. The DNI was the difference between the leukocyte differentials assayed in the MPO channel and that assayed in the nuclear lobularity channel. The complete blood cell (CBC) count is frequently and routinely evaluated in pregnant women with PPROM [9]. Therefore, DNI values can be easily calculated without any additional evaluations. In this study, the DNI in the PPROM without HCA group showed a value of 0 (range, 0–2.6) and the DNI in the PPROM with HCA group showed a higher value of 0.25 (0–4.7) (*p* = 0.006). In addition, we evaluated the DNI value of those pregnant women without PPROM or chorioamnionitis who delivered healthy neonates after 37 gestational weeks. The DNI value in the normal pregnant group was 0 (range, 0–2). In the logistic regression analysis, the odds ratio of the DNI was 1.818 (95% CI: 1.104–2.991, *p* = 0.018) and the odds ratio of the CRP level was 1.069 (95% CI: 1.017–1.125, *p* = 0.009). The odds ratio was higher for the DNI than for the prevalently used CRP level. The DNI can also increase in various infectious conditions [9, 10, 28]. Therefore, it shows low sensitivity and a positive predictive value for diagnosing HCA in patients with PPROM. Thus, instead of using each individual marker alone, we established an equation model to improve the predictive value of HCA.

Many researchers have shown that the NLR is an independent diagnostic parameter for systemic inflammation and stress conditions, owing to the fact that leukocytes play a major role in these conditions through an increase in neutrophils and decrease in lymphocytes [29, 30]. In this study, the neutrophil count in the PPROM with HCA group increased to 7.57 × 10^9^/L (range, 3.05–22.62 × 10^9^/L) and that in the PPROM without HCA group increased to 8.67 × 10^9^/L (3.42–23.41 × 10^9^/L); however, the difference was not significant (*p* = 0.114). The lymphocyte count slightly decreased in the PPROM with HCA group, and this result is similar to that reported previously. The NLR is being increasingly used as a predictive marker for spontaneous preterm birth and intrauterine infection in preterm delivery [21, 31]. The NLR increased in the PPROM with HCA group, but only the lymphocyte count showed a relatively high difference between the two groups. Therefore, only the lymphocyte count was included in the predictive equation. Multiple previous studies have reported that corticosteroid treatment during pregnancy can cause a transient increase in the total WBC count, including neutrophils [32–34]. In our study, 31.0% (27/87) of patients in the PPROM without HCA group and 40.3% (25/62) of patients in the PPROM with HCA group had undergone corticosteroid treatment between PPROM and birth. In the PPROM with HCA group, only one patient had undergone corticosteroid treatment once before being transferred to our hospital. Excluding this one patient, corticosteroid treatment was administered after blood sampling for the DNI assessment in the remaining patients. Thus, there is a low possibility of corticosteroid treatment affecting the WBC and neutrophil counts.

CRP is an acute-phase protein produced by the liver, and it is widely used as an objective marker for detecting tissue damage and infection. As the CRP level increases in chorioamnionitis or neonatal sepsis conditions, it is used in the obstetrical field to detect infectious conditions. Previous studies reported that an increasing CRP level was highly associated with preterm delivery and poor perinatal outcomes in pregnant women with preterm labor or PPROM [35–37].

The present study has some limitations. The DNI can only be calculated by measuring immature granulocytes using the ADIVA 2120 Hematology System. However, there are several hematology analyzers that can measure immature granulocytes, such as the Sysmex XE-series hematology analyzer (Sysmex Corporation, Kobe, Kansia, Japan) and Cell-Dyn series hematology analyzer (Abbot Diagnostics, Abbott Park, III). Although the measurement methods for immature granulocytes differ depending on the analyzer used, we could not identify any differences in the immature granulocyte values. Additionally, we conducted this study using only the DNI values obtained at the time when the patients with PPROM were admitted to the hospital. Therefore, changes in the DNI values are still unclear according to the course of PPROM. In addition, we were not able to prove the correlation between the severity of HCA and the predictive equation due to the unclear recording of the HCA stages in the pathologic results. Thus, a well-designed prospective study is needed in order to evaluate the usefulness of the DNI as a predictive marker of HCA.

In conclusion, the serum DNI, a new inflammation parameter, could be a predictive marker for chorioamnionitis or funisitis in patients with PPROM. Our predictive equation involving maternal serum markers could be used as a non-invasive, inexpensive, and simple method for detecting the placental inflammatory response in women with PPROM.
